# *In silico* characterization of IncX3 plasmids carrying *bla*_OXA-181_ in *Enterobacterales*

**DOI:** 10.3389/fcimb.2022.988236

**Published:** 2022-09-08

**Authors:** Zhijian Yu, Zhengrong Zhang, Lile Shi, Shengni Hua, Ting Luan, Qiuping Lin, Zhixiong Zheng, Xiaosan Feng, Mubiao Liu, Xiaobin Li

**Affiliations:** ^1^ Department of Otolaryngology, Zhuhai People’s Hospital, Zhuhai Hospital Affiliated with Jinan University, Zhuhai, China; ^2^ Department of Urology, Zhuhai People’s Hospital, Zhuhai Hospital Affiliated with Jinan University, Zhuhai, China; ^3^ Department of Cardiology, Zhuhai People’s Hospital, Zhuhai Hospital Affiliated with Jinan University, Zhuhai, China; ^4^ Department of Radiation Oncology, Zhuhai People’s Hospital, Zhuhai Hospital Affiliated with Jinan University, Zhuhai, China; ^5^ Community Health Service Center of Xinkou Town, Tianjin, China; ^6^ Zhuhai Precision Medical Center, Zhuhai People’s Hospital, Zhuhai Hospital Affiliated with Jinan University, Zhuhai, China; ^7^ Department of Neonatology, Zhuhai People’s Hospital, Zhuhai Hospital Affiliated with Jinan University, Zhuhai, China; ^8^ Department of Obstetrics and Gynecology, Zhuhai People’s Hospital, Zhuhai Hospital Affiliated with Jinan University, Zhuhai, China

**Keywords:** *Enterobacterales*, plasmid, *bla*_OXA-181_, conjugative transfer region, genetic context

## Abstract

Carbapenem-resistant *Enterobacterales* poses a global urgent antibiotic resistance threat because of its ability to transfer carbapenemase genes to other bacteria *via* horizontal gene transfer mediated by mobile genetic elements such as plasmids. Oxacillinase-181 (OXA-181) is one of the most common OXA-48-like carbapenemases, and OXA-181-producing *Enterobacterales* has been reported in many countries worldwide. However, systematic research concerning the overall picture of plasmids harboring *bla*_OXA-181_ in *Enterobacterales* is currently scarce. In this study, we aimed to determine the phylogeny and evolution of *bla*_OXA-181_-positive (gene encoding OXA-181) plasmids. To characterize the plasmids harboring *bla*_OXA-181_ in *Enterobacterales*, we identified 81 *bla*_OXA-181_-positive plasmids from 35,150 bacterial plasmids downloaded from the NCBI RefSeq database. Our results indicated that diverse plasmid types harbored *bla*_OXA-181_ but was predominantly carried by IncX3-type plasmids. We systematically compared the host strains, plasmid types, conjugative transfer regions, and genetic contexts of *bla*_OXA-181_ among the 66 *bla*_OXA-181_-positive IncX3 plasmids. We found that IncX3 plasmids harboring *bla*_OXA-181_ were mostly ColKP3-IncX3 hybrid plasmids with a length of 51 kb each and were mainly distributed in *Escherichia coli* and *Klebsiella pneumoniae*. Most of the IncX3 plasmids harboring *bla*_OXA-181_ were human origin. Almost all the *bla*_OXA-181_-positive IncX3 plasmids were found to carry genes coding for relaxases of the MOB_P_ family and VirB-like type IV secretion system (T4SS) gene clusters, and all the 66 IncX3 plasmids were found to carry the genes encoding type IV coupling proteins (T4CPs) of the VirD4/TraG subfamily. Most IncX3 plasmids harbored both *bla*_OXA-181_ and *qnrS1* in their genomes, and the two antibiotic resistance genes were found to a composite transposon bracketed by two copies of insertion sequence IS*26* in the same orientation. Our findings provide important insights into the phylogeny and evolution of *bla*_OXA-181_-positive IncX3 plasmids and further address their role in acquiring and spreading *bla*_OXA-181_ genes in *Enterobacterales*.

## Introduction

The rapid increase in carbapenemase-producing *Enterobacterales* has become a public-health threat (Kim et al., 2021). Surveillance studies have shown that OXA-48-like carbapenemases are the most common carbapenemases in *Enterobacterales* in certain regions of the world (Pitout et al., 2019). Oxacillinase-181 (OXA-181) is a carbapenem-hydrolyzing class D β-lactamase, a variant of OXA-48 differing by four amino acid substitutions, possessing a higher ability to hydrolyze carbapenems (Potron et al., 2011; Oueslati et al., 2015). The OXA-181 was first reported in clinical carbapenem-resistant *Klebsiella pneumoniae* (*K. pneumoniae*) and *Enterobacter cloacae* strains in Indian hospitals in 2007 (Castanheira et al., 2011). Since then, OXA-181-producing *Enterobacterales*, mainly *K. pneumoniae* and *Escherichia coli* (*E. coli*), have been reported in several countries worldwide (Balm et al., 2013; Liu et al., 2015; Rojas et al., 2017; Piazza et al., 2018; Mouftah et al., 2019), indicating a trend of increasing prevalence.

*Enterobacterales* cause both hospital- and community-acquired infections (Rood and Li, 2017). Carbapenem-resistant *Enterobacterales* (CRE) has now emerged worldwide as an urgent antibiotic resistance threat because these bacteria can transfer carbapenemase genes to other bacteria *via* horizontal gene transfer mediated by mobile genetic elements such as plasmids (Nordmann et al., 2011). Antimicrobial resistance (AMR) in CRE isolates is frequently encoded by plasmid-borne genes, and can disseminate clonally or horizontally (Rozwandowicz et al., 2018). Four different plasmid types belonging to the ColKP3, IncX3, IncN1, and IncT replicon types have been reported to harbor OXA-181 gene (*bla*_OXA-181_) (Pitout et al., 2019).

Conjugative plasmids are important vehicles for the dissemination of antibiotic resistant genes (ARGs) (Smillie et al., 2010; Ravi et al., 2018). These plasmids typically have the conserved backbone regions and the variable accessory regions (Brown et al., 2013; Sitter et al., 2021). The former contains genes encoding plasmid-related traits, such as replication control and conjugation functions, while the latter have accessory genes, such as genes encoding antibiotic resistance, which are usually located on the transposons or integrons (Norman et al., 2009; Norberg et al., 2011). However, systematic research on the backbone and accessory regions of plasmids harboring *bla*_OXA-181_ in *Enterobacterales* is currently scarce.

In this study, we performed *in silico* typing and comparative analysis of *bla*_OXA-181_-positive plasmids from 35,150 bacterial plasmids downloaded from the NCBI RefSeq database. We analyzed and compared the host strains, plasmid replicon types, conjugative transfer regions, and genetic contexts of the *bla*_OXA-181_ gene among the *bla*_OXA-181_-positive plasmids. This study provides important insights into the phylogeny and evolution of *bla*_OXA-181_-positive plasmids and further addresses their role in the acquisition and spread of ARGs.

## Materials and methods

### Plasmid genomic sequences

RefSeq database at NCBI (O’Leary et al., 2016) is a comprehensive, integrated, non-redundant, well-annotated set of reference sequences. *Via* the FTP release directory “Plasmid (https://ftp.ncbi.nih.gov/refseq/release/plasmid/),” we accessed and downloaded all the plasmids available in the RefSeq database. A total of 35,150 bacterial plasmid genomic sequences were downloaded on July 14, 2021 from the NCBI RefSeq database (**Table S1**). The genome data (FASTA DNA format) were downloaded in batches using two Bioperl modules including Bio::DB::GenBank and Bio::SeqIO.

### Identification of bacterial plasmids harboring *bla*_OXA-181_

The β-lactamase genes within the genomes of plasmids were identified in the 35,150 plasmids using the ResFinder software version 4.1 (https://cge.cbs.dtu.dk/services/ResFinder/) (Bortolaia et al., 2020), with a minimum coverage of 60% and minimum identity of 90%. The term “*bla*_OXA-181_” was used to search in the “Resistance gene” list within the ResFinder results to determine *bla*_OXA-181_-positive plasmids.

### Plasmid replicon typing of the *bla*_OXA-181_-positive plasmids

Plasmid replicon typing was performed using the PlasmidFinder software (https://cge.cbs.dtu.dk/services/PlasmidFinder/) (Carattoli and Hasman, 2020). Selecting the database “*Enterobacterales*”, the DNA files in FASTA format were analyzed in batches using the PlasmidFinder software, with minimum coverage of 60% and minimum identity of 95%.

### Phylogenetic analyses of the *bla*_OXA-181_-positive plasmids

The files of the *bla*_OXA-181_-positive plasmids identified by ResFinder in GenBank format were downloaded in batches using the Bio::DB::GenBank and Bio::SeqIO modules. Files containing protein sequences were extracted from the files in GenBank format using the Bioperl/Bio::SeqIO module. For all *bla*_OXA-181_-positive plasmids, phylogenetic patterns based on the presence/absence of orthologous gene families were analyzed. A binary protein presence/absence matrix was created using OrthoFinder (http://www.stevekellylab.com/software/orthofinder) (Emms and Kelly, 2019) with DIAMOND for sequence similarity searches, and then a hierarchical cluster result was generated by PAST3 (Hammer et al., 2001) and eventually displayed by iTOL (https://itol.embl.de/) (Letunic and Bork, 2016).

### Characterization of the conjugative regions of *bla*_OXA-181_-positive plasmids

To determine the presence/absence of *oriT*s, relaxase genes, T4CP genes, and gene cluster for T4SS, the files in GenBank format of the *bla*_OXA-181_-positive plasmids were analyzed in batches using the software oriTfinder (https://bioinfo-mml.sjtu.edu.cn/oriTfinder/) (Li et al., 2018) (local version). Furthermore, the types of *oriT*s, relaxase genes, and T4CP genes were identified based on the exhibition of oriTDB database (https://bioinfo-mml.sjtu.edu.cn/oriTDB/index.php) (Li et al., 2018). In addition, the types of gene clusters for T4SS were classified based on the SecReT4 database (https://bioinfo-mml.sjtu.edu.cn/SecReT4/) (Bi et al., 2013).

### Genetic context analysis towards the *bla*_OXA-181_ and other ARGs

The bacterial insertion sequences within the *bla*_OXA-181_-positive plasmids in *Enterobacterales* were explored using ISfinder software (Siguier et al., 2006). Comparisons among the genetic contexts of *bla*_OXA-181_ were performed using BLAST Ring Image Generator (BRIG) (Alikhan et al., 2011).

## Results

### General characteristics of *bla*_OXA-181_-positive plasmids

**Figure 1:**
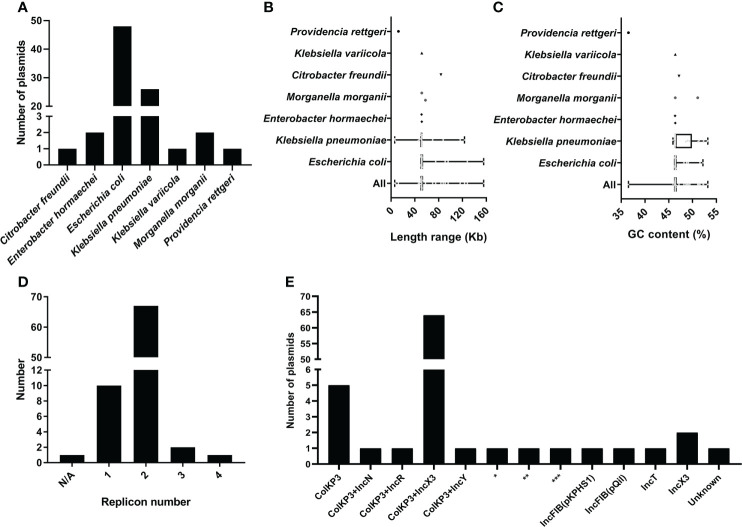
Characteristics of 81 *bla*_OXA-181_-positive plasmids in *Enterobacterales*. **(A)** Histogram about number of plasmids distributed in different species for the 81 *bla*_OXA-181_-positive in *Enterobacterales*. **(B)** Length distribution of the *bla*_OXA-181_-positive plasmids in different species. **(C)** GC content distribution of the *bla*_OXA-181_-positive plasmids in different species. **(D)** Histogram of number of replicons per plasmid for the 81 *bla*_OXA-181_-positive plasmids. **(E)** Histogram of number of combination modes of different replicons among the 81 *bla*_OXA-181_-positive plasmids. *representing ColKP3+IncFIB(pB171)+IncFII; **representing ColKP3+IncFIB(AP001918)+IncFIC(FII); ***representing ColKP3+IncFIA+IncFIB(AP001918)+IncFII(pRSB107).

Using ResFinder, 81 plasmids bearing *bla*_OXA-181_ (**Table S2**) were identified from 35150 bacterial plasmids downloaded from the NCBI RefSeq database. By analyzing the taxonomy of the bacterial strains containing the *bla*_OXA-181_-positive plasmids, we found that the 81 plasmids were distributed in seven different species (**Figure 1A**). The predominant species carrying *bla*_OXA-181_-positive plasmids was *E. coli*, accounting for 59.26% (48 *bla*_OXA-181_-positive plasmids), followed by *K. pneumoniae*, accounting for 30.10% (26 *bla*_OXA-181_-positive plasmids). Other species with *bla*_OXA-181_-positive plasmids were *Enterobacter hormaechei* (two plasmids), *Morganella morganii* (two plasmids), *Citrobacter freundii* (one plasmid), *Klebsiella variicola* (one plasmid), and *Providencia rettgeri* (one plasmid). Overall, all the strains harboring *bla*_OXA-181_-positive plasmids belonged to the order *Enterobacterales*, including family *Enterobacteriaceae* and family *Morganellaceae*.

We analyzed and compared the genome sizes of the *bla*_OXA-181_-positive plasmids, and compared the sizes of the *bla*_OXA-181_-positive plasmids distributed in different species. The genome sizes of the 81 *bla*_OXA-181_-positive plasmids varied from 6.103 kb to 155.5 kb, with the 25th percentile, median, and 75th percentile being 51.47 kb, 51.48 kb, and 51.48 kb, respectively (**Figure 1B**). The sizes of the 48 *bla*_OXA-181_-positive plasmids in *E. coli* varied from 50.14 kb to 155.5 kb (25th percentile = 51.48 kb; 75th percentile = 51.48 kb), with a median size of 51.48 kb. The sizes of the 26 *bla*_OXA-181_-positive plasmids in *K. pneumoniae* varied from 6.103 kb to 123.3 kb (25th percentile = 49.50 kb; 75th percentile = 51.48 kb), with a median size of 51.48 kb.

We calculated the GC contents of the 81 *bla*_OXA-181_-positive plasmids. The GC contents of the 81 *bla*_OXA-181_-positive plasmids ranged from 36.51% to 53.20% (25th percentile = 46.37%; 75th percentile = 46.38%), with a median GC content of 46.37% (**Figure 1C**). The GC contents of the 48 *bla*_OXA-181_-positive plasmids of *E. coli* varied from 46.22% to 52.19% (25th percentile = 46.37%; 75th percentile = 46.38%), with a median GC content of 46.37%. The GC contents of the 26 *bla*_OXA-181_-positive plasmids of *K. pneumoniae* varied from 45.88% to 53.20% (25th percentile = 46.37%; 75th percentile = 49.87%), with a median GC content of 46.37%.

### Replicon types in plasmids harboring *bla*_OXA-181_

Among the 81 *bla*_OXA-181_-bearing plasmids, 80 were successfully identified their replicon types, including 10 single-replicon plasmids and 70 multi-replicon plasmids (67 plasmids with two replicons, two plasmids with three replicons, and one plasmid with four replicons) (**Figure 1D**). Of the 67 plasmids with two replicons, 64 plasmids were ColKP3-IncX3 hybrid plasmids (**Figure 1E**). Of the 10 single-replicon plasmids, five plasmids had ColKP3 replicon (**Figure 1E**). Notably, 75 of the 81 *bla*_OXA-181_-positive plasmids in our study carried ColKP3 replicon.

### Identification of the *bla*_OXA-181_-positive conjugative IncX3 plasmids

**Figure 2:**
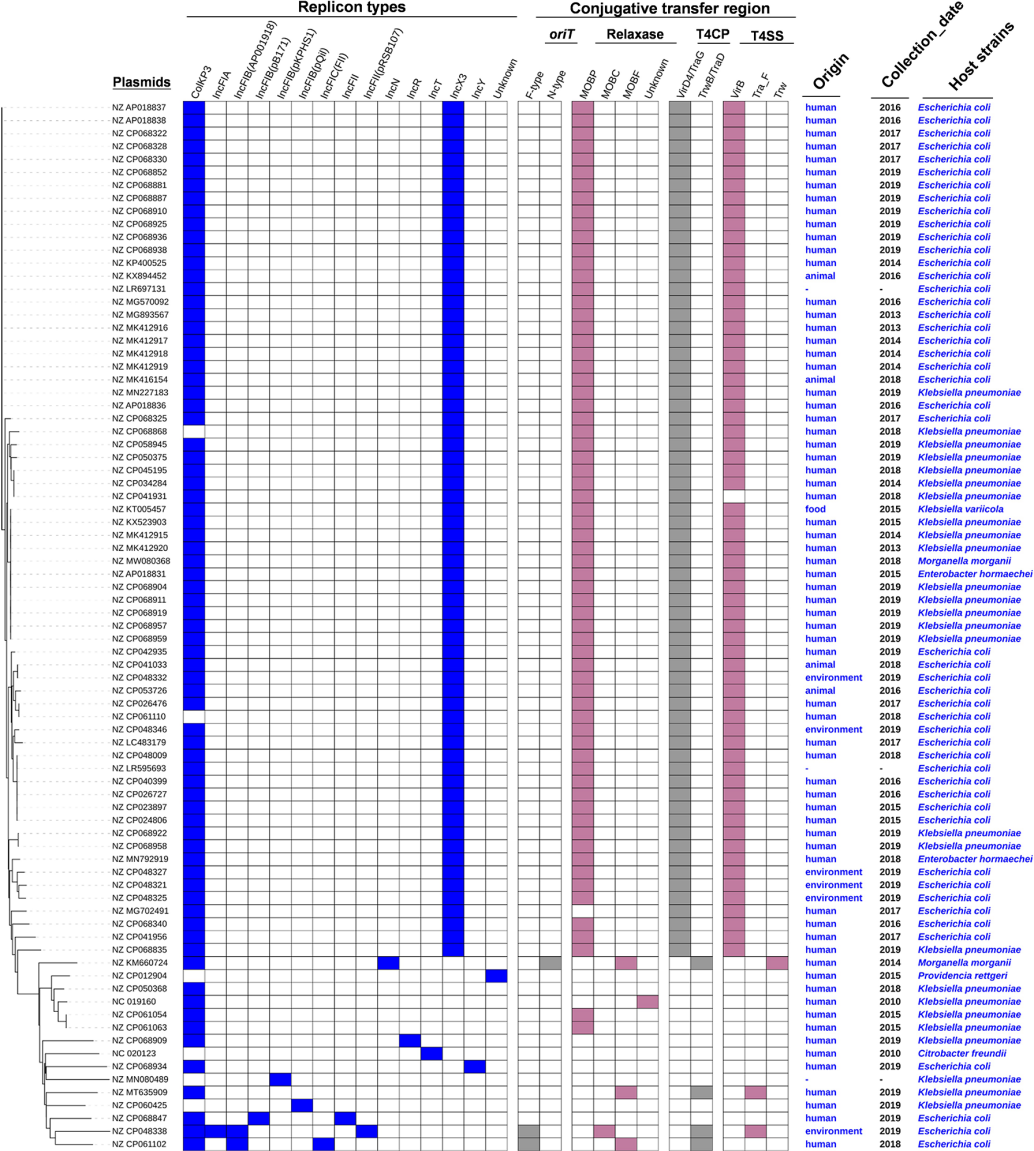
Details of replicon types, conjugative transfer regions, and host strains of the 81 *bla*_OXA-181_-positive plasmids in *Enterobacterales*. The six categories of information present in this figure include the phylogenetic tree, replicon types, conjugative transfer regions (*oriT*, relaxase, T4CP, and T4SS), origin, collection date, and host strains of 81 *bla*_OXA-181_-positive plasmids.

**Figure 3:**
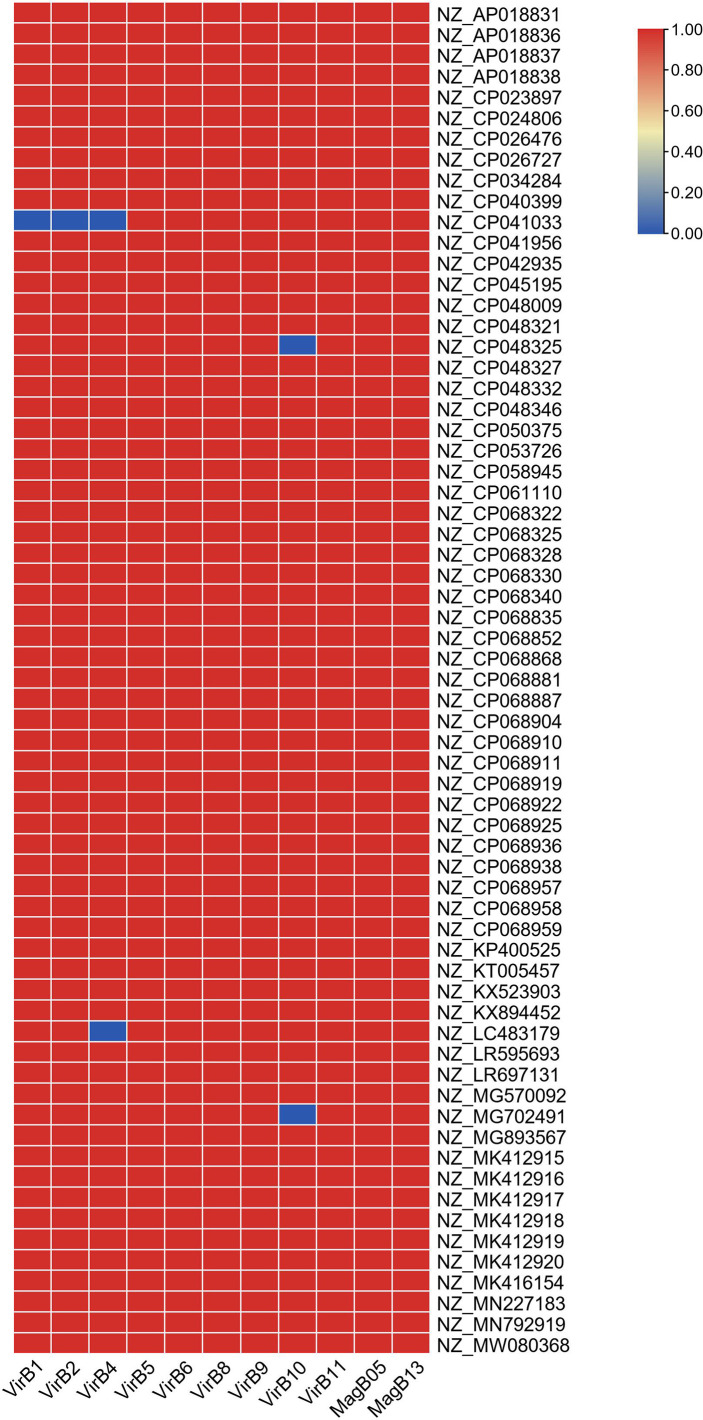
Details of the VirB-like T4SS gene clusters in the 66 *bla*_OXA-181_-positive IncX3 plasmids in *Enterobacterales*. Red/blue colors indicate gene presence/absence.

To obtain a comprehensive overview of *bla*_OXA-181_-positive plasmids, we constructed phylogenetic trees (**Figure 2**). Based on the phylogenetic patterns, replicon types, and conjugative transfer regions, the 81 *bla*_OXA-181_-positive plasmids were classified into two main clades. One clade contained 66 *bla*_OXA-181_-positive plasmids (81.48%) and represented the most common conjugative plasmid pattern carrying *bla*_OXA-181_ gene. Of the 66 *bla*_OXA-181_-positive plasmids, 65 carried genes encoding relaxases of the MOB_P_ family. All the 66 *bla*_OXA-181_-positive plasmids harbored genes encoding type IV coupling proteins (T4CPs) of the VirD4/TraG subfamily. In addition, 65 of the 66 *bla*_OXA-181_-positive plasmids contained VirB-like type IV secretion system (T4SS) gene clusters (**Figures 2**, **3**). Most of the VirB-like T4SS gene clusters were composed of 11 core genes (**Figure 3**). The current version of oriTfinder could not identify the *oriT* sites of the clade; however, 354-bp intergenic sequences flanking the relaxase genes were identified as *oriT*–like regions and had the inverted repeat (IR) sequence “TAACTA..TAGTTA” (**Figure S1**). Of the 66 *bla*_OXA-181_-positive conjugative IncX3 plasmids, 64 plasmids were identified with two replicons: ColKP3 and IncX3, and the other two plasmids were single-replicon IncX3 plasmids.

### General characteristics of the *bla*_OXA-181_-positive conjugative IncX3 plasmids

In this study, the *bla*_OXA-181_-positive conjugative IncX3 plasmids were mainly distributed in the strains of *E. coli* (44 plasmids) and *K. pneumoniae* (18 plasmids) (**Figure 2** and **Figure S2**). Overall, all the *bla*_OXA-181_-positive conjugative IncX3 plasmids included 65 *bla*_OXA-181_-positive conjugative plasmids belonging to family *Enterobacteriaceae* and one plasmid belonging to family *Morganellaceae* (**Figure 2** and **Figure S2**).

Of the 66 *bla*_OXA-181_-harboring IncX3 plasmids, 54 were human origin, accounting for 81.82% (**Figure 2** and **Table S3**). In addition, four *bla*_OXA-181_-harboring IncX3 plasmids were found to be animal origin, five IncX3 plasmids harboring *bla*_OXA-181_ were animal origin, and one *bla*_OXA-181_-bearing IncX3 plasmid was food origin (**Figure 2** and **Table S3**).

**Figure 4:**
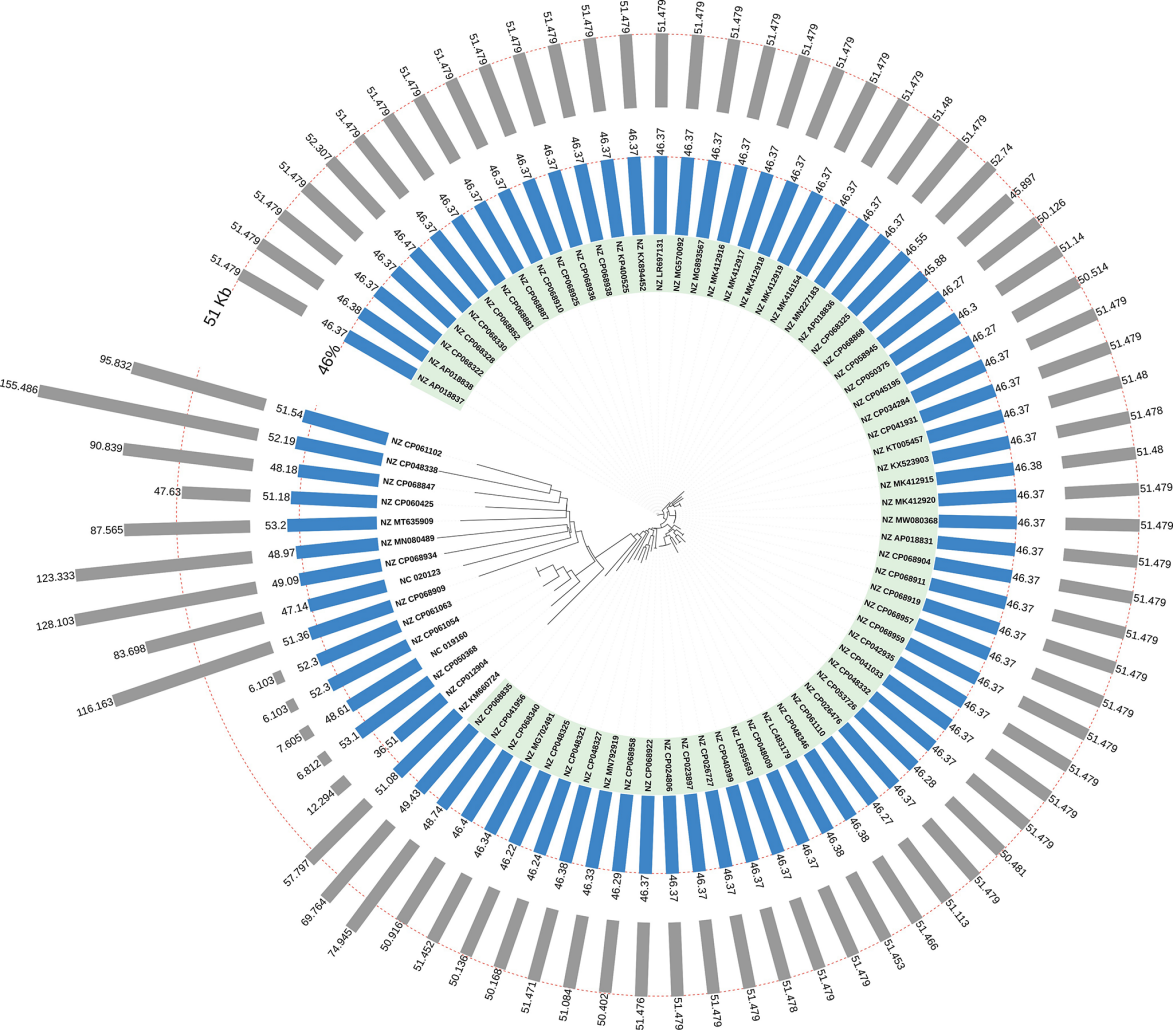
Distribution of lengths and GC contents of the 81 *bla*_OXA-181_-harboring plasmids in *Enterobacterales*. The 66 *bla*_OXA-181_-harboring IncX3 plasmids were highlighted in light green. The outermost grey bars denote the lengths of the *bla*_OXA-181_-harboring plasmids. The bars in light blue denote the GC content of the 81 *bla*_OXA-181_-harboring plasmids.

We analyzed and compared the genome sizes of all the 66 *bla*_OXA-181_-harboring conjugative plasmids. The genome sizes of 66 *bla*_OXA-181_-positive IncX3 plasmids varied from 45.90 kb to 74.95 kb (25th percentile = 51.48 kb; 75th percentile = 51.48 kb), with the median size of 51.48 kb (**Figure 4**). The genome sizes of the 44 *bla*_OXA-181_-positive conjugative IncX3 plasmids in *E. coli* ranged from 45.14 kb to 74.95 kb (25th percentile = 51.48 kb; 75th percentile = 51.48 kb), with a median size of 51.48 kb (**Figure 4**). The genome sizes of the 18 *bla*_OXA-181_-positive conjugative IncX3 plasmids in *K. pneumoniae* ranged from 45.90 kb to 69.76 kb (25th percentile = 50.98 kb; 75th percentile = 51.48 kb), with a median of 51.48 kb (**Figure 4**).

### Co-existence of *bla*_OXA-181_ and *qnrS1* within *bla*_OXA-181_-positive conjugative IncX3 plasmids

Among the 66 *bla*_OXA-181_-positive IncX3 plasmids, 63 were found to carry two acquired ARGs: *bla*_OXA-181_ and *qnrS1* in their genomes; the former encoded OXA-181-type carbapenemase and the latter was responding for resistance to quinolone (**Figure S3**). Two *bla*_OXA-181_-positive IncX3 plasmids from *K. pneumoniae* (*K. pneumoniae* strain BA39649 plasmid pColKP3_IncX3 with GenBank accession number of NZ_CP058945 and *K. pneumoniae* strain RIVM_C017275 plasmid pRIVM_C017275_2 with GenBank accession number of NZ_CP068868) only contained the *bla*_OXA-181_ gene (**Figure S3**). In the *E. coli* strain EC2 IncX3 plasmid pEC2_1 (NZ_CP041956), in addition to the *bla*_OXA-181_ and *qnrS1*, six other acquired ARGs were also identified, including *bla*_CTX-M-15_, *bla*_TEM-1B_, *bla*_OXA-1_, *aac(6’)-Ib-cr*, *catB3*, and *tet(A)* (**Figure S3**). In the *K. pneumoniae* strain RIVM_C018652 IncX3 plasmid pRIVM_C018652_2 (NZ_CP068835), eight acquired ARGs [*mph(A)*, *bla*_TEM-1B_, *rmtB*, *bla*_NDM-5_, *sul1*, *aadA2*, *dfrA12*, and *bla*_OXA-181_] were found but no *qnrS1* was present in its genome (**Figure S3**).

### Genetic contexts associated with *bla*_OXA-181_ and *qnrS1* in the *bla*_OXA-181_-positive conjugative IncX3 plasmids

**Figure 5:**
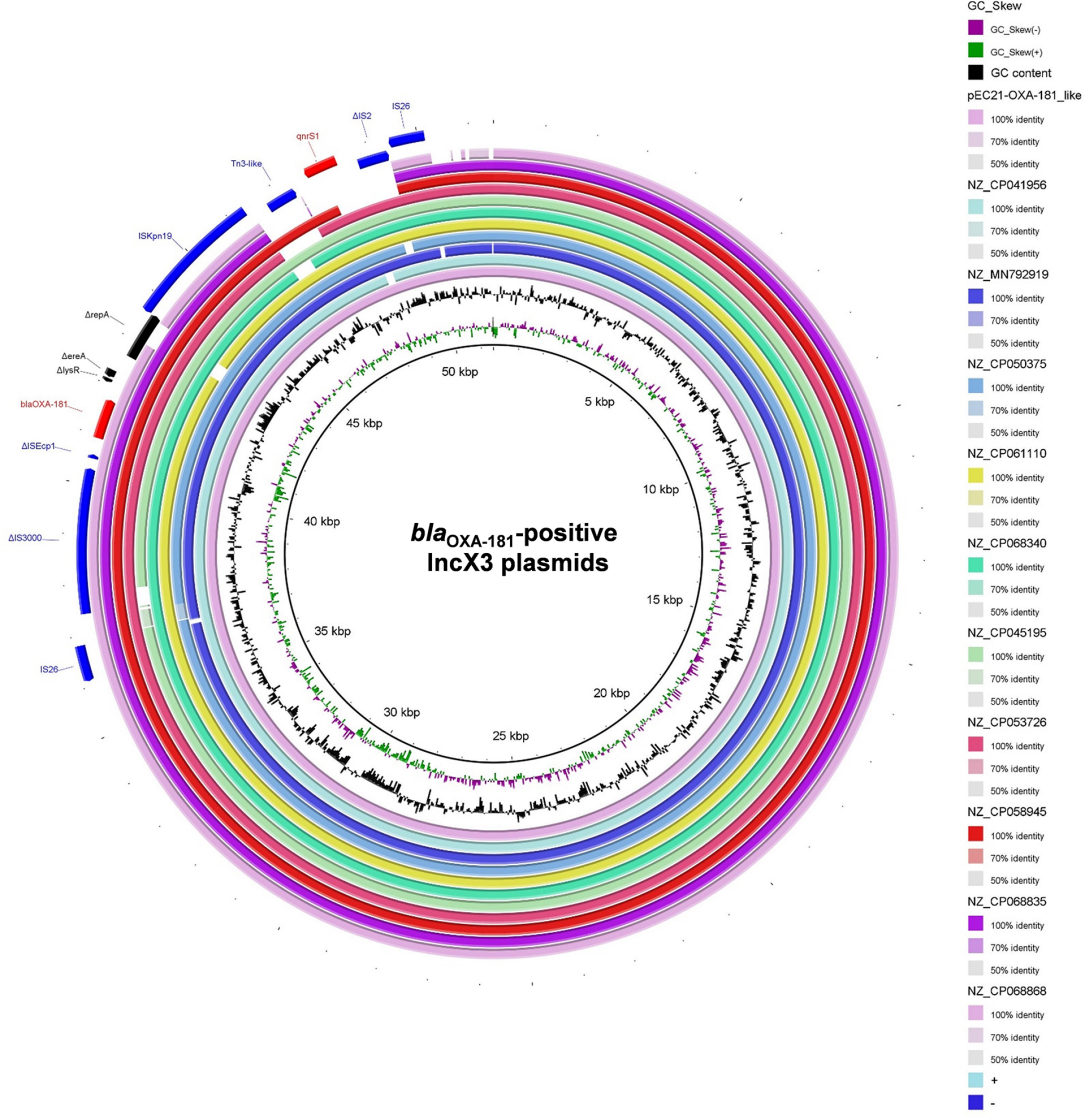
Schematic map of *bla*_OXA-181_-associated genetic structures identified among the 66 *bla*_OXA-181_-positive IncX3 plasmids in *Enterobacterales*. The pEC21-OXA-181_like represent the 56 plasmids carrying the intact IS*26*-flanked composite transposon.

In the accessory regions of the 63 IncX3 plasmids harboring both *bla*_OXA-181_ and *qnrS1* in their genomes, the *bla*_OXA-181_ and *qnrS1* were located in a composite transposon, which was bracketed by two copies of insertion sequence IS*26* in the same orientation (**Table S4** and **Figure 5**). Of the 63 IncX3 plasmids with both *bla*_OXA-181_ and *qnrS1*, 56 were found to carry almost identical genetic contexts associated with *bla*_OXA-181_ and *qnrS1*, the intact IS*26*-flanked composite transposon (**Table S4**). Ten *bla*_OXA-181_-positive IncX3 plasmids were found to carry the truncated IS*26*-flanked composite transposon (**Figure 5**).

The immediate genetic environment of *bla*_OXA-181_ (IS*26*–ΔIS*3000*–ΔIS*Ecp1*–*bla*_OXA-181_–Δ*lysR*–Δ*ere*–Δ*repA*–IS*Kpn19*) were found to situated in all the 66 *bla*_OXA-181_-positive IncX3 plasmids. For the immediate genetic environment of *qnrS1*, the *qnrS1* was flanked by a Tn*3*-like transposon (truncated by an IS*Kpn19*) and an IS*2*-like insertion sequence (truncated by an IS*26*) (**Figure 5**).

## Discussion

OXA-181 is one of the most common OXA-48-like derivative, and the OXA-181-producing *Enterobacterales* has been reported in many countries worldwide (Pitout et al., 2019). To characterize the plasmids harboring *bla*_OXA-181_ in *Enterobacterales*, we identified and analyzed 81 *bla*_OXA-181_-positive plasmids, which were selected from 35,150 bacterial plasmids collected from all over the world. Our results show that diverse plasmid types harbor *bla*_OXA-181_ but IncX3 plasmids, especially the ColKP3-IncX3 hybrid plasmids, predominantly carry it, indicating the potential of IncX3 plasmids as vehicles in the global dissemination of OXA-181. IncX3 plasmids are narrow-host range plasmids in *Enterobacterales* (Johnson et al., 2012), which have been reported to carry various carbapenemase genes in CRE worldwide (Mouftah et al., 2019). To further characterize the IncX3 plasmids harboring *bla*_OXA-181_, we systematically compared the plasmid types, conjugative transfer regions, as well as the genetic features associated with *bla*_OXA-181_ in the 66 *bla*_OXA-181_-positive IncX3 plasmids.

The common species bearing the *bla*_OXA-181_-positive IncX3 plasmids were *E. coli* (44 plasmids) and *K. pneumoniae* (18 plasmids). OXA-181 was first described for *K. pneumoniae* (Castanheira et al., 2011; Potron et al., 2011). *K. pneumoniae* represents one of the most concerning pathogens known for its high frequency and diversity of AMR genes (Navon-Venezia et al., 2017; Wyres and Holt, 2018), and it has been classified as an ESKAPE organism (De Oliveira et al., 2020). Herein, we found that *K. pneumoniae* was the second most prevalent species harboring the *bla*_OXA-181_-positive IncX3 plasmids. In our study, *E. coli* was the predominant species carrying the *bla*_OXA-181_-positive IncX3 plasmids. AMR in *E. coli* has become an issue of concern in both human and veterinary health worldwide (Poirel et al., 2018).

Our work indicated that almost all the IncX3 plasmids harboring *bla*_OXA-181_ were ColKP3-IncX3 hybrid plasmids. ColKP3-type plasmid was also reported to harbor *bla*_OXA-232_, another gene encoding OXA-48-like carbapenemase (Shu et al., 2019). By comparing the sizes of IncX3 plasmids harboring *bla*_OXA-181_, we found that the OXA-181-encoding gene *bla*_OXA-181_ was mostly located in 51-kb IncX3-type plasmids. A previous study has demonstrated that *bla*_OXA-181_-positive IncX3 plasmid, 51-kb pOXA181_EC14828 in *E. coli* ST410 from China, was a self-transmissible plasmid, as it could be transferred to the recipient strain in the conjugation experiment (Liu et al., 2015).

Conjugative plasmids play a central role in facilitating horizontal genetic exchange and therefore promote the acquisition and spread of AMR genes (Partridge et al., 2018; Jiang et al., 2020). The conjugative transfer regions of plasmids typically consist of four modules: *oriT* region, relaxase gene, T4CP gene, and gene cluster for T4SS apparatus (de la Cruz et al., 2010). Herein, we attempted to analyze and compare the conjugative transfer regions located in the *bla*_OXA-181_-positive IncX3 plasmids in *Enterobacterales* using the software oriTfinder (Li et al., 2018). Almost all the IncX3 plasmids harboring *bla*_OXA-181_ contained genes coding for relaxases belonging to the MOB_P_ family characterized by the domain “Relaxase (Pfam: PF03432)”. The TraI encoded by the IncPα plasmid RP4 (Pansegrau et al., 1993) is regarded as a representative of MOB_P_ family. T4CPs are essential elements in conjugative T4SSs and are also key elements in many pathogenic T4SSs (Álvarez-Rodríguez et al., 2020). In this study, all the 66 *bla*_OXA-181_-positive plasmids in *Enterobacterales* carried the genes encoding T4CPs of the VirD4/TraG subfamily characterized by the domain “T4SS-DNA_transf (Pfam: PF02534)”. Almost all *bla*_OXA-181_-positive IncX3 plasmids contained VirB-like T4SS gene clusters, which are by far the best characterized T4SS (Guglielmini et al., 2014). Notably, we cannot identify the *oriT* sites in *bla*_OXA-181_-positive IncX3 plasmids using oriTfinder, but the 354-bp intergenic sequences flanking the relaxase genes were *oriT*–like regions.

In our study, we found that 63 of the 66 IncX3 plasmids harbored both *bla*_OXA-181_ and *qnrS1* in their genomes. The two acquired ARGs were found to be embedded in a composite transposon, which is bracketed by two copies of the insertion sequence IS*26* oriented in the same direction. The IS*26* has been shown to play a major role in the dissemination of ARGs in gram-negative bacteria (Harmer and Hall, 2021) because IS26 can recruit ARGs into the mobile gene pool by forming transposons carrying many different resistance genes (Harmer and Hall, 2016). The *bla*_OXA-181_ harbored by all the 66 IncX3 plasmids was adjacent to IS*Ecp1*. The IS*Ecp1* is a member of the IS*1380* family known to mobilize adjacent DNA sequences by a so-called one-ended transposition mechanism (Poirel et al., 2005), and it has been shown to be involved in mobilization of different antibiotic resistance genes such as *bla*_CTX-M_ (Poirel et al., 2003). The truncation of the IS*Ecp1* adjacent to *bla*_OXA-181_ in the 66 *bla*_OXA-181_-positive IncX3 plasmids suggested that the IS*26*-flanked composite transposon has the potential to mobilize *bla*_OXA-181_ independent of the action of IS*Ecp1*.

## Conclusion

In this study, we identified 81 *bla*_OXA-181_-harboring plasmids from 35,150 bacterial plasmids downloaded from the NCBI RefSeq database. Diverse plasmid types harbored *bla*_OXA-181_ but was predominantly carried by IncX3-type plasmids. Our study mainly focused on *in silico* characterization of the 66 *bla*_OXA-181_-bearing IncX3 plasmids, including host strains, plasmid types, origin, conjugative transfer regions, and genetic contexts. We found that IncX3 plasmids harboring *bla*_OXA-181_ were mostly ColKP3-IncX3 hybrid plasmids with a length of 51 kb each and were mainly distributed in *Escherichia coli* and *Klebsiella pneumoniae*. Most of the IncX3 plasmids harboring *bla*_OXA-181_ were found to be human origin. For the conjugative transfer regions, almost all the *bla*_OXA-181_-positive IncX3 plasmids were found to carry genes coding for relaxases of the MOB_P_ family and VirB-like type IV secretion system (T4SS) gene clusters, and all the 66 IncX3 plasmids were found to carry the genes encoding type IV coupling proteins (T4CPs) of the VirD4/TraG subfamily. Plasmid analysis revealed that *bla*_OXA-181_ together with the quinolone resistance gene *qnrS1* were carried by an IS*26*-flanked composite transposon on the IncX3-type plasmids. Our findings enhance our understanding of the genetic diversity and characteristics of *bla*_OXA-181_-harboring IncX3 plasmids and further address their role in acquiring and spreading *bla*_OXA-181_ genes in *Enterobacterales*.

## Data availability statement

The datasets presented in this study can be found in online repositories. The names of the repository/repositories and accession number(s) can be found in the article/**Supplementary Material**.

## Author contributions

XL, ML and XF designed the study. XL and ZY analysed all the data and drafted the manuscript. SH, TL, QL, and ZXZ contributed to the data acquisition and methodology. ZRZ and LS reviewed and edited the manuscript. All authors contributed to the article and approved the submitted version.

## Funding

This work was supported financially by the National Natural Science Foundation of China (Grant No. 81902460 and 82002170), the Guangdong Basic and Applied Basic Research Foundation (Grant No. 2019A1515011725), the Xiangshan Talent Project of Zhuhai People’s Hospital (Grant No. 2020XSYC-02), the Cultivation Project of Zhuhai People’s Hospital (2019PY-19 and 2019PY-22).

## Conflict of interest

The authors declare that the research was conducted in the absence of any commercial or financial relationships that could be construed as a potential conflict of interest.

## Publisher’s note

All claims expressed in this article are solely those of the authors and do not necessarily represent those of their affiliated organizations, or those of the publisher, the editors and the reviewers. Any product that may be evaluated in this article, or claim that may be made by its manufacturer, is not guaranteed or endorsed by the publisher.
