# Supplementary material for: In silico characterization of IncX3 plasmids carrying bla OXA-181 in Enterobacterales
Source: Front Cell Infect Microbiol. 2022 Sep 8;12:988236. doi: 10.3389/fcimb.2022.988236 (PMC9492964; doi:10.3389/fcimb.2022.988236)
Supplement: Supplementary file 1 [file DataSheet_1.docx]

***In Silico* Characterization of IncX3 Plasmids** **Carrying *bla*_OXA-181_ in *Enterobacterales***

**Table legends：**

**Table S1**. Information of 35,150 plasmids downloaded from the NCBI RefSeq database.

**Table S2**. Acquired antibiotic resistant genes of 81 plasmids bearing *bla*_OXA-181_ identified using the Resfinder.

**Table S3**. Clinical data of the 81 plasmids bearing *bla*_OXA-181_ in *Enterobacterales*.

**Table S4**. Results of alignments with the genetic contexts associated with *bla*_OXA-181_ and *qnrS1* (coordinate: 36109..50135) of plasmid pEC21-OXA-181 in *E. coli* strain EC21 and other 65 *bla*_OXA-181_-positive IncX3 plasmids in *Enterobacterales*.

**Figure legends:**

**Figure S1**. Conjugative transfer regions (including the *oriT*-like region) of the *E. coli* strain EC21 plasmid pEC21-OXA-181 (NZ_MG893567).

**Figure S2**. Histogram about number of plasmids distributed in different species for the 66 *bla*_OXA-181_-positive IncX3 plasmids in *Enterobacterales*.

**Figure S3**. An overview of the ResFinder-facilitated detection of acquired antimicrobial resistance genes (ARGs) towards the 81 plasmids bearing *bla*_OXA-181_ gene. The plasmids marked by light-green were the 66 *bla*_OXA-181_-positive IncX3 plasmids in *Enterobacterales.*


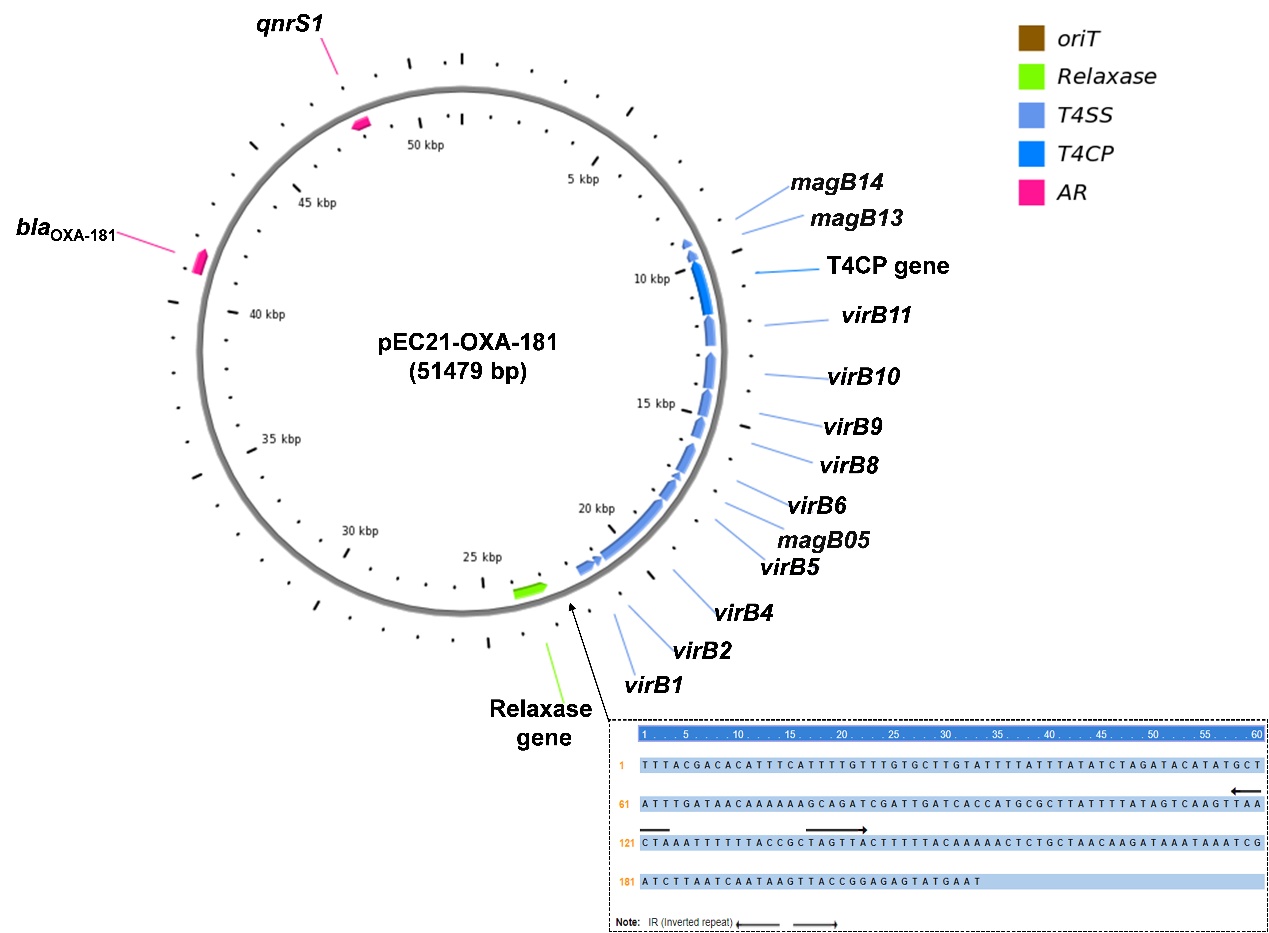


Figure S1


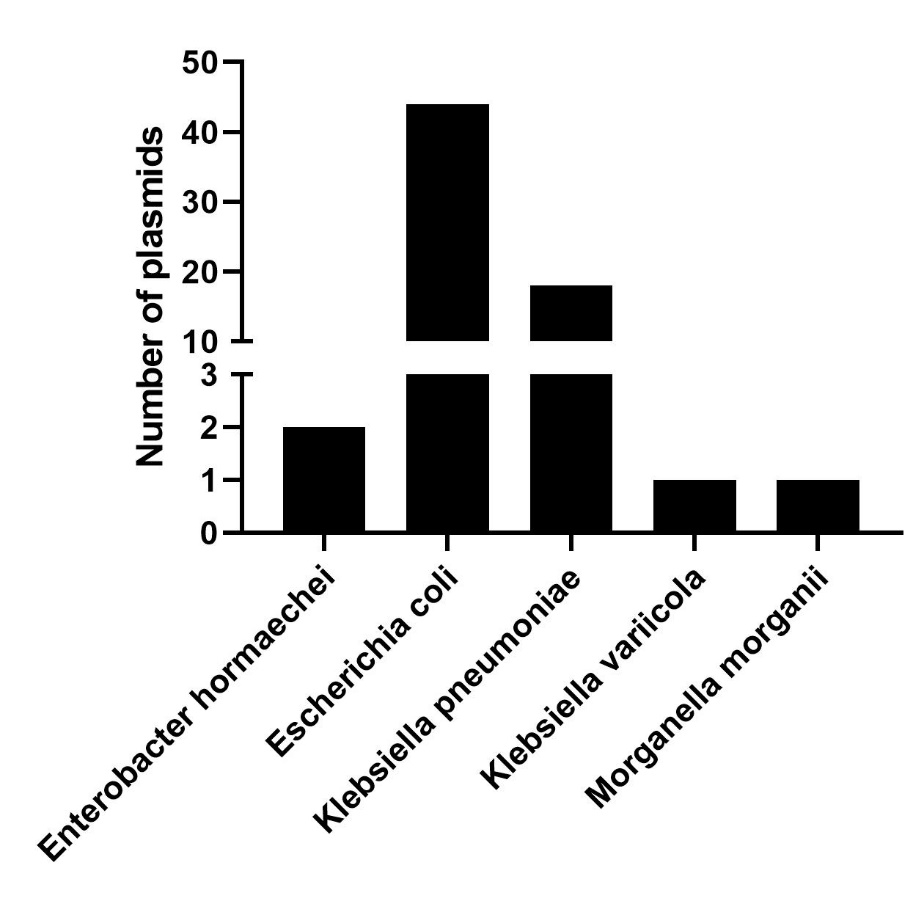


Figure S2


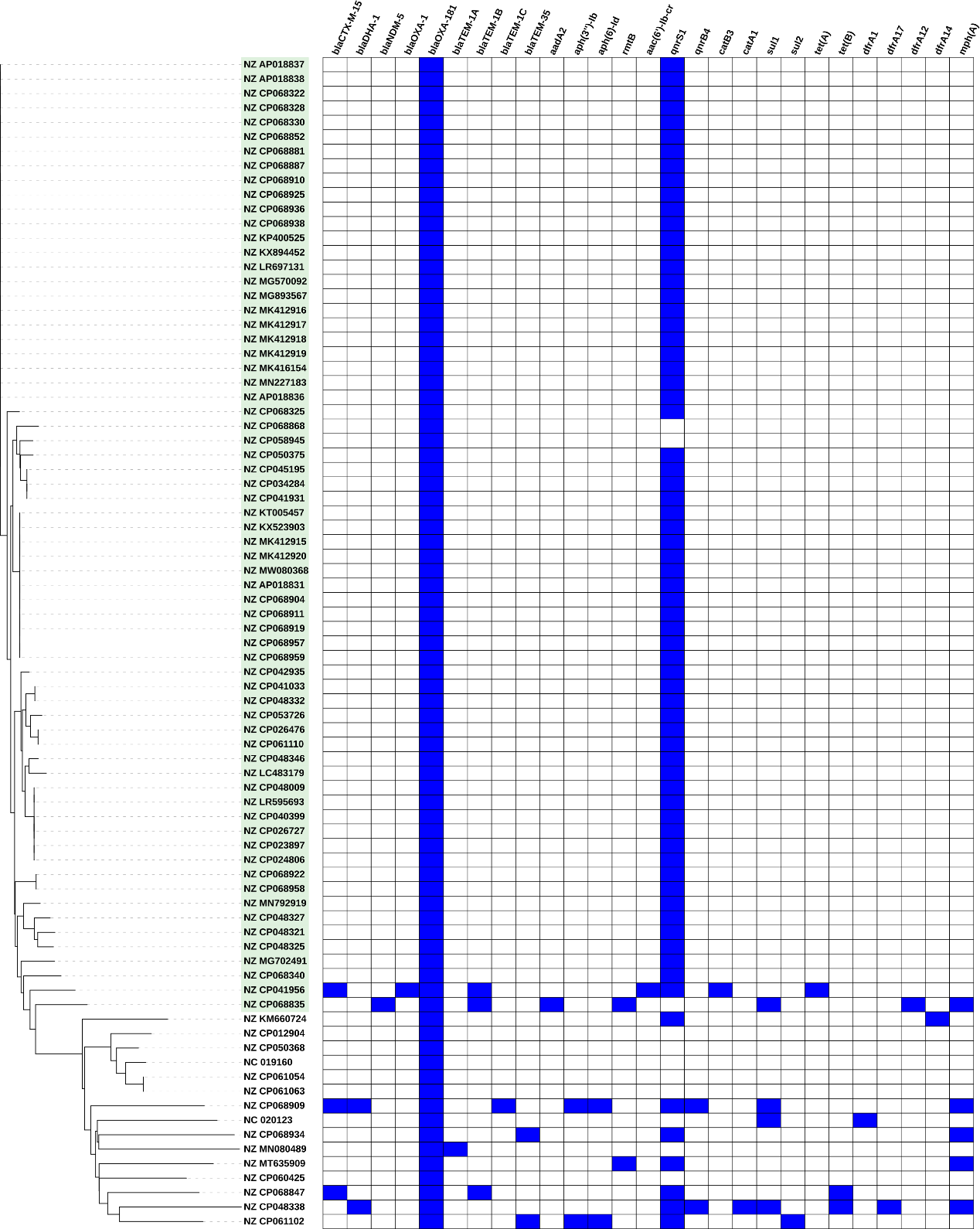


Figure S3
